# The microRNA-29 family in cartilage homeostasis and osteoarthritis

**DOI:** 10.1007/s00109-015-1374-z

**Published:** 2015-12-19

**Authors:** Linh T. T. Le, Tracey E. Swingler, Natalie Crowe, Tonia L. Vincent, Matthew J. Barter, Simon T. Donell, Anne M. Delany, Tamas Dalmay, David A. Young, Ian M. Clark

**Affiliations:** Biomedical Research Centre, School of Biological Sciences, Norwich Research Park, University of East Anglia, Norwich, Norfolk NR4 7TJ UK; Department of Cell Signalling, Kennedy Institute of Rheumatology, University of Oxford, Oxfordshire, UK; Musculoskeletal Research Group, Institute of Cellular Medicine, Newcastle University, Newcastle-upon-Tyne, UK; Institute of Orthopaedics, Norfolk and Norwich University Hospital, Norfolk, UK; Center for Molecular Medicine, University of Connecticut Health Center, Farmington, CT USA

**Keywords:** MicroRNA, Osteoarthritis, Sry-related box 9, Chondrocyte, Wnt pathway

## Abstract

**Abstract:**

MicroRNAs have been shown to function in cartilage development and homeostasis, as well as in progression of osteoarthritis. The objective of the current study was to identify microRNAs involved in the onset or early progression of osteoarthritis and characterise their function in chondrocytes. MicroRNA expression in mouse knee joints post-DMM surgery was measured over 7 days. Expression of miR-29b-3p was increased at day 1 and regulated in the opposite direction to its potential targets. In a mouse model of cartilage injury and in end-stage human OA cartilage, the miR-29 family was also regulated. SOX9 repressed expression of miR-29a-3p and miR-29b-3p via the 29a/b1 promoter. TGFβ1 decreased expression of miR-29a, b, and c (3p) in primary chondrocytes, whilst IL-1β increased (but LPS decreased) their expression. The miR-29 family negatively regulated Smad, NFκB, and canonical WNT signalling pathways. Expression profiles revealed regulation of new WNT-related genes. Amongst these, *FZD3*, *FZD5*, *DVL3*, *FRAT2*, and *CK2A2* were validated as direct targets of the miR-29 family. These data identify the miR-29 family as microRNAs acting across development and progression of OA. They are regulated by factors which are important in OA and impact on relevant signalling pathways.

**Key messages:**

- Expression of the miR-29 family is regulated in cartilage during osteoarthritis.
- SOX9 represses expression of the miR-29 family in chondrocytes.
- The miR-29 family is regulated by TGF-β1 and IL-1 in chondrocytes.
- The miR-29 family negatively regulates Smad, NFκB, and canonical Wnt signalling.
- Several Wnt-related genes are direct targets of the miR-29 family.

**Electronic supplementary material:**

The online version of this article (doi:10.1007/s00109-015-1374-z) contains supplementary material, which is available to authorized users.

## Introduction

MicroRNAs (miRNAs) are short endogenous non-protein-coding RNA molecules, typically 19–25 nucleotides in length [1]. They mediate the post-transcriptional regulation of protein-coding genes by binding to the 3’ untranslated region (3’ UTR) of target mRNAs leading to translational inhibition or mRNA degradation, depending on the degree of sequence complementarity. MicroRNAs can be master regulators of gene expression and influence virtually all cell activities and events [2]. As of January 2015, a total of 1881 precursors and 2588 mature human miRNAs have been annotated in miRBase release 21.0 [3]. Their function and regulation, however, are still only poorly understood.

Osteoarthritis (OA) is a degenerative joint disease characterised by degradation of articular cartilage, thickening of subchondral bone and osteophyte formation. The aetiology of OA is complex with a number of factors contributing to the disease: genetic, environmental, developmental, biochemical and biomechanical [4]. Estimates are that over 10 % of the world population above 60 years old have OA, with a large socioeconomic burden [5]. There are no effective disease-modifying drugs to treat OA, and drugs that relieve pain are often insufficient, with joint replacement offered to patients at end-stage disease. Increasing age and obesity are major risk factors for OA, and changing population demographics suggests that the burden of disease will rise [4].

Recent evidence reveals miRNAs as prominent modulators of OA pathogenesis. MicroRNA-140, originally identified as a cartilage- and skeletal-restricted miRNA in zebrafish [6] and mouse development [7], is currently the best described miRNA in cartilaginous tissue. Universal knockout of miR-140 lead to mild craniofacial deformities and dwarfism [8, 9]. These mice prematurely develop OA-like changes with age and are more susceptible to OA in the ‘destabilisation of the medial meniscus’ (DMM) model, whilst transgenic mice overexpressing miR-140 in cartilage were resistant to antigen-induced arthritis. A number of miRNAs are reported to be altered in expression between late-stage OA cartilage and their normal articular counterpart and/or in cartilage development [10]. Since miR-140 has such a key role in cartilage homeostasis and osteoarthritis, it is likely that other miRNAs will show similar function as this is explored.

Sry-related box 9 (Sox9) is a transcription factor and master regulator of cartilage formation, required for multiple steps in the differentiation of chondrocytes [11]. It regulates expression of both type II collagen and proteoglycan, markers of the chondrocyte phenotype [12]. Several miRNAs are regulated by Sox9 (e.g. miR-140 and miR-455) [13–15] or regulate Sox9 expression (e.g. miR-675 and miR-145) [16, 17].

We performed miRNA and mRNA profiling in total RNA purified from whole mouse knee joints at early time points after DMM surgery to identify important miRNAs in the development of OA. Among these modulated miRNAs, the expression of miR-29b-3p, a member of miR-29 family, was increased at 1 day post-surgery when it was regulated in the opposite direction to its potential targets. The miR-29 family in human and mouse consists of miR-29a, miR-29b (b1 and b2 which are identical mature miRNAs) and miR-29c, with the mature microRNAs differing only in two or three bases. The family is encoded at two human genomic loci on chromosome 1 (encoding a primary transcript for miR-29a and miR-29b1) and chromosome 7 (encoding a primary transcript for miR-29b2 and miR-29c). The family is known to regulate a number of extracellular matrix genes, as well as regulatory targets [18].

Hence, we investigated the expression of the miR-29 family in a number of models relevant to cartilage and OA and their regulation and function in chondrocytes.

## Materials and methods

### Destabilization of the medial meniscus (DMM) model

C57BL/6 mice were purchased from Harlan, UK. Animal experiments were performed following ethical and statutory approval in accordance with local policy. Mice were maintained at 21 °C in standard, individually ventilated cages holding 3–6 mice per cage. Ten-week-old male mice were anaesthetized by inhalation of Isoflurane (3 % induction and 1.5–2 % maintenance) in 1.5–2 L/min oxygen. All animals received a subcutaneous injection of Vetergesic (Alstoe Animal Heath Ltd) post-surgery. The mice were fully mobile within 4–5 min following withdrawal of Isoflurane. Destabilization of the medial meniscus (DMM) was performed as previously described [19] with capsulotomy only as sham [20]. The contralateral (left) knees were non-operated controls. Knees were harvested at 1, 3 and 7 days post-surgery.

### Patient samples

Femoral head and knee cartilage were obtained from osteoarthritis (hip OA (HOA) age 61–89 years, four female and two male; knee OA (KOA) age 55–71 years, two female and three male) and trauma (NOF (neck of femur), age 71–92 years, four female and two male) patients undergoing total joint replacement surgery at the Norfolk and Norwich University Hospital. OA was diagnosed using clinical history and examination, coupled with X-ray findings; confirmation of gross pathology was made at time of joint removal. Fracture patients had no known history of joint disease, and their cartilage was free of lesions. This study was performed with Ethical Committee approval, and all patients provided informed consent.

### Cell culture

SW1353 chondrosarcoma cells were from the American Type Culture Collection and cultured in Dulbecco’s modified Eagle’s medium (DMEM, Life Technologies) containing 10 % (*v*/*v*) fetal bovine serum (Sigma), 2 mM glutamine, 100 IU/ml penicillin and 100 μg/ml streptomycin as described [14]. Primary human articular chondrocytes (HACs) were isolated from cartilage of osteoarthritis patients as described [21] and cultured as above. DF1 cells (a gift from Prof. Andrea Münsterberg, University of East Anglia, UK) are spontaneously transformed chicken embryo fibroblast cells [22] and were cultured as above. Human mesenchymal stem cells (hMSC) were resuspended in chondrogenic culture medium consisting of high glucose Dulbecco’s modified Eagle’s medium containing 100 μg/ml sodium pyruvate (Lonza), 10 ng/ml TGF-β3 (Peprotech), 100 nM dexamethasone, 1× ITS-1 premix, 40 μg/ml proline and 25 μg/ml ascorbate-2-phosphate (all from Sigma-Aldrich, Poole, UK). About 5 × 10^5^ hMSC in 100 μl medium were pipetted onto 6.5-mm diameter, 0.4-μm pore size polycarbonate Transwell filters (Merck Millipore), centrifuged in a 24-well plate (200 g, 5 min), then 0.5 ml of chondrogenic medium was added to the lower well as described [15]. Media were replaced every 2 or 3 days up to 14 days. Induction of the murine chondrogenic cell line, ATDC5 was as described in [14]. For micromass culture, primary HACs were grown in monolayer culture until passage two. Confluent cells were then trypsinised and resuspended at a density of 2 × 10^7^ cells/ml in DMEM high glucose, with GlutaMAX (Life Technologies), containing 10 % (*v*/*v*) fetal bovine serum, 100 IU/ml penicillin and 100 μg/ml streptomycin (growth medium). Micromass was obtained by pipetting 20 ul cell suspension into individual wells of 24-well plates and incubating for 3 h to attach. One ml growth medium was then added, and the micromass was incubated for 24 h. Growth medium was then replaced with DMEM high glucose medium containing 1× insulin-transferrin-selenium (Life Technologies), with or without cytokines or growth factors for 48 h. Inhibition of NFκB was achieved using JSH-23 (Calbiochem) and inhibition of p38 MAP kinase used SB203580 (Sigma) at the concentrations shown.

### Mouse femoral head cartilage wounding assay

The mouse femoral head cartilage wounding assay was as described [23]. Tissue was harvested into 500 μl Trizol (Life Technologies) at the time points: 0, 1, 3, 6, 12, 24 and 48 h and stored at −80 °C.

### RNA isolation and qRT-PCR

Trizol reagent was used to isolate total RNA from cultured cells according to manufacturer’s instructions; a further purification step using the mirVana miRNA Isolation Kit (Life Technologies) was performed for total RNA from cartilage. The miRCURY LNA™ Universal cDNA synthesis kit (Exiqon, Denmark) and miRNA-specific LNA™ primers (Exiqon) were used for quantification of mature miRNA transcripts by qRT-PCR. Data were normalized to U6 as the housekeeping gene. For mRNA, SuperScript III RT (Life Technologies) using 1 μg of total RNA (Dnase I treated) with random hexamers (Life Technologies) was used with data normalised to expression of 18S ribosomal RNA. Primer sequences are listed in Supplementary Table 1. Both miRNA and mRNA relative quantifications were calculated using the ∆∆C_T_ method. Fluorescence for each cycle was analysed by the real-time PCR 7500 system (Applied Biosystems).

### Microarray analyses

For miRNA analysis in the DMM model, for each time point, total RNA samples from four animals were pooled for array. MicroRNA microarray was performed by Exiqon (Denmark). The raw data were background corrected, normalized by the locally weighted scatterplot smoothing (LOWESS) algorithm and summarized at the log_2_-scale using the program R. Profiling of mRNA used the Illumina Human HT12v4 platform or Mouse WG-6 platform (Source Bioscience). Data were imported to R Studios and analysed using the Bioconductor packages. Expression values were normalized using Robust Multichip Average (RMA) methodology. The 3’ UTR sequences of mRNAs were imported from Ensembl into R. The miRNA seed sites were identified using the BioStrings package. Frequency of mRNAs which are targets of a miRNA at each fold change value was calculated as the ratio of mRNA containing a seed site to the total number of mRNAs modulated with that fold change.

### Transient tranfection

The 3’ UTR of mRNAs containing the predicted binding site of miR-29-3p were subcloned into pmirGLO (Promega), using QuikChange (Agilent) to introduce mutations. Constructs were sequence verified. DF1 cells were seeded into a 96-well plate at 3.75 × 10^4^ cells/cm^2^ overnight and transiently transfected with 100 ng reporter plasmid, 50 nM miRNA mimic, inhibitor or control using Lipofectamine 2000 according to manufacturer’s instructions (Life Technologies) and incubated for 24 or 48 h. Cell lysates were assayed for luciferase using the Dual Luciferase Reporter Assay Kit (Promega), read with an EnVision 2103 Multilabel plate reader (Perkin Elmer). Relative luciferase activity was the ratio of firefly luciferase to Renilla luciferase activity. Signalling pathways were measured using p(CAGA)_12_-luc (Smad2/3) [24]; κB-luc (NFκB) [25] and TOPFlash (canonical Wnt) [26]. One hundred nanogram of the plasmid and 10 ng of constitutive Renilla plasmid were co-transfected into SW1353 cells with 50 nM miR-29 mimic, inhibitor or control. After serum starvation for 24 h, cells were treated with recombinant human TGFβ1 (4 ng/ml), IL-1β (5 ng/ml), or Wnt3a (100 ng/ml) (R&D Systems) for 6 h. For promoter assays, 100 ng of plasmid containing the miR-29a/b1 promoter [27] was co-transfected with 100 ng of human SOX9 expression plasmid (a gift of Dr. Simon Tew, University of Liverpool, UK, [28]) or control plasmid, and 10 ng of Renilla plasmid in SW1353 cells for 24 h. Knockdown of SOX9 used 100 nM SOX9 small interfering RNA (siRNA) (Dharmacon) for 48 h. For loss- or gain-of-function experiments, primary HACs were plated at 2.5 × 10^5^ cells per well of a 6-well plate overnight. Cells were transfected for 6 h in serum- and antibiotic-free DMEM using Lipofectamine 2000 (Invitrogen), miR-29b-3p mimic at 30 nM (Qiagen), miR-29b-3p inhibitor at 50 nM (Qiagen), or non-targeting controls (All Stars at 30 nM (Qiagen), miScript Inhibitor control at 50 nM (Qiagen)). Media were replaced with complete medium for a further 48 h prior to harvest.

### Statistical analysis

Data were analysed using Student’s *t* test to compare between two samples or one-way ANOVA with post hoc Tukey’s test to compare between multiple samples using GraphPad Prism version 5.

## Results

### Expression of the microRNA-29 family in osteoarthritis

In order to identify the specific miRNAs involved in osteoarthritis, we explored the expression of miRNAs early after surgery in a murine model of joint destabilisation. This was reinforced by measuring expression in a murine model of cartilage injury and in human cartilage from end-stage OA.

**Fig. 1:**
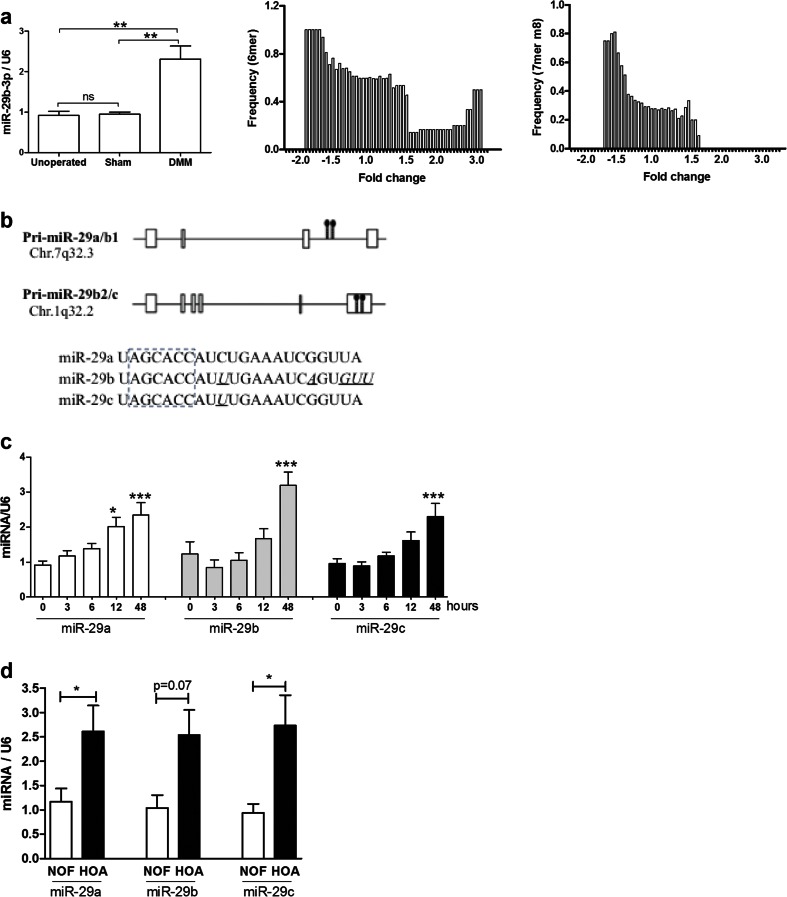
Identification of the miR-29 family in osteoarthritis. **a** MicroRNA-29b-3p was measured by qRT-PCR from RNA obtained from mouse knee joints at 24 h post-surgery after ‘destabilisation of the medial meniscus’ (mean ± SEM, ANOVA, *n* = 3). The frequency of predicted miR-29 targets in mRNA with seed sites of either 6mer or 7mer m8 was calculated (see Material and Methods) across fold changes of gene expression. **b** In man, the miR-29 family is expressed from two loci, with the primary miR-29a/b1 on chromosome 7 (the last intron of the primary transcript GenBank accession EU154353) and primary miR-29b2/c on chromosome 1 (the last exon of the primary transcript GenBank accession numbers EU154351 and EU154352) [29]; mature sequences of miR-29a, **b** and **c** (3p) have identical seed sequences. **c** The miR-29 family was measured by qRT-PCR in the murine hip avulsion injury model over 48 h post-injury (mean ± SEM, ANOVA, *n* = 6). *Empty bars*, miR-29a; *grey bars*, miR-29b and *black bars*, miR-29c. **d** The miR-29 family was measured by qRT-PCR in cartilage from hip osteoarthritis (HOA, *filled bars*) patients compared to fracture (NOF, *empty bars*) controls (qRT-PCR, mean ± SEM, Student’s *t* test, *n* = 6). Data were normalised to U6 RNA expression *p < 0.05; **p < 0.01; ***p < 0.001

In the murine DMM model, total RNA was isolated from whole joints at day 1, 3 and 7 post-surgery and pools of four replicates subjected to miRNA and mRNA microarray. Comparing the operated knee with contralateral control showed two miRNAs increased by DMM surgery >1.5-fold at day 1 (miR-144-3p and miR-29b-3p) and two miRNAs at day 3 (miR-370-5p and miR-21-5p). Greater than 30 miRNAs altered expression >1.5-fold at day 7 (Supplementary Table 2). Expression of mRNAs showed the same pattern of greater change at day 7. Correlation analysis between miRNA and their putative mRNA targets showed that for the four miRNAs with altered expression at day 1 or 3 post-surgery, there was an inverse correlation only between miR-29b-3p and its putative targets (genes containing either 6mer or 7mer-m8 seed sites). Of the genes whose expression was decreased when expression of the miRNA was increased, there was an enrichment of potential miR-29-3p targets identified and vice versa (Fig. 1a). Since miRNAs function by decreasing their target mRNAs, this suggests functional involvement of this miRNA at the early stages of the DMM model. Expression of miR-29b-3p was validated by qRT-PCR in the knee undergoing both DMM and sham surgery compared to un-operated control at day 1 post-surgery (Fig. 1a). MicroRNA-29b is a member of the miR-29 family, along with miR-29a and 29c, which are encoded at two genomic loci and share a seed sequence (Fig. 1b) [29]; all were included in downstream investigations since their seed sites are identical.

Cartilage injury can precede OA [30], and a murine cartilage injury model (hip avulsion) was shown to display similar gene expression patterns to the DMM model [23]. Measurement of the miR-29-3p family in this model showed a significant increase in expression at 12–48 h post-explantation of cartilage, with a trend to increase for miR-29a and c at 6 h (Fig. 1c).

Expression of the miR-29 family was measured in human articular cartilage from hip replacement surgery for OA or fracture to the neck-of-femur (NOF, control). The expression of miR-29a-3p, miR-29b-3p and miR-29c-3p was increased in osteoarthritis compared to NOF (Fig. 1d).

### Expression of the microRNA-29 family in chondrocyte differentiation and regulation by SOX9

**Fig. 2:**
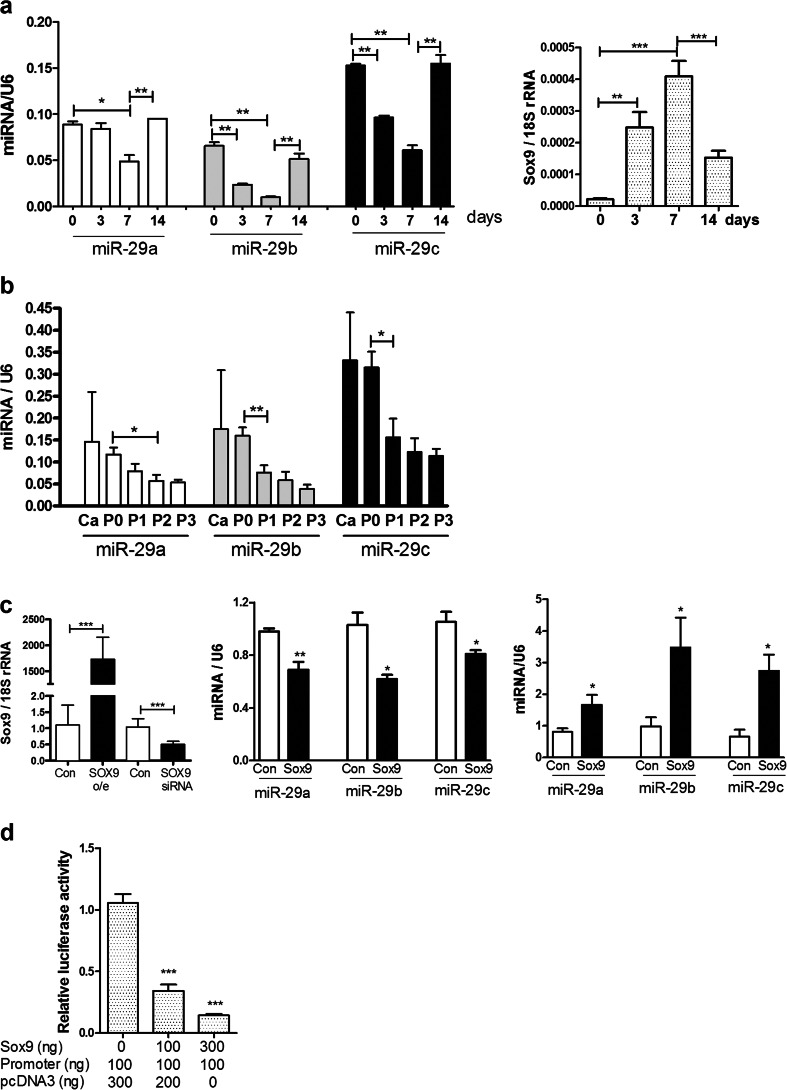
Expression of the miR-29 family in chondrocyte differentiation. Expression of the miR-29 family and SOX9 was measured by qRT-PCR from RNA **a** obtained from human mesenchymal stem cells induced through chondrogenesis to form cartilage discs at day 0, 3, 7 and 14 (mean ± SEM, ANOVA, *n* = 6); *empty bars*, miR-29a; *grey bars*, miR-29b; *black bars*, miR-29c. **b** purified from osteoarthritic knee cartilage tissue (Ca) or isolated from chondrocytes in monolayer culture at P0, P1, P2 and P3 (mean ± SEM, ANOVA, *n* = 4 for cartilage and *n* = 8 for cultured chondrocytes). Data were normalised to U6 RNA expression. *Empty bars*, miR-29a; *grey bars*, miR-29b; *black bars*, miR-29c. **c** A SOX9 expression vector or empty pcDNA3 vector (control), SOX9 siRNA or a non-targeting control (control) were transiently transfected into SW1353 for 48 h. SOX9 was measured by qRT-PCR and normalised to 18S rRNA expression (mean ± SEM, Student’s *t* test, *n* = 3 (overexpression), *n* = 5 (siRNA). Expression of the miR-29 family was measured by qRT-PCR after overexpression or knockdown of SOX9; data were normalised to U6 RNA expression (mean ± SEM, Student’s *t* test, *n* = 3 (overexpression), *n* = 4 (siRNA). *Empty bars*, control; *filled bars*, Sox9 overexpression or knockdown. **d** A primary miR-29a/b1 promoter-reporter construct was transfected into SW1353 cells with the empty pcDNA3 vector to equalise DNA transfected, luciferase activity was measured at 24 h and data were normalised to Renilla luciferase from a co-transfected constitutive expression vector (mean ± SEM, ANOVA, *n* = 6). *p < 0.05; **p < 0.01; ***p < 0.001. Data were normalised to U6 RNA expression

Since the replay of chondrogenesis and altered cell differentiation can contribute to OA [31], expression of the miR-29 family was investigated in appropriate models. In human MSC differentiation to form cartilage discs over 14 days, the miR-29 family decreased in expression to 7 days, returning to starting levels by 14 days (Fig. 2a). This pattern was inversely correlated with SOX9 expression (Fig. 2a). A similar pattern was seen in the murine ATDC5 chondrogenesis model over 42 days, with decrease to 21 days, returning to baseline by 42 days (data not shown). In the human model, the expression profile of both miRNA and mRNA was measured using array as described [15]. An enrichment of potential miR-29 targets was identified in upregulated genes, when expression of the miR-29 family was low (data not shown) suggesting functional involvement of the miR-29 family.

Expression of the miR-29 family was also measured across dedifferentiation of human articular chondrocytes upon serial passage in monolayer culture. Expression decreased with subculture (Fig. 2b) with a similar pattern to COL2A1 [32].

Sox9 is a critical transcription factor regulating chondrocyte differentiation [33]. From data above, we hypothesised that SOX9 was a negative regulator of miR-29 expression in chondrocytes. Overexpression of SOX9 in SW1353 cells leads to a decrease in expression of the miR-29 family, whilst knockdown of SOX9 increased their expression (Fig. 2c). The 2 kb region upstream of the miR-29a/b1 transcription start site contains five putative binding sites for SOX9 (data not shown). Co-transfection of this region in a promoter-reporter plasmid with a SOX9 expression construct showed a dose-dependent decrease in luciferase activity with increasing SOX9 expression (Fig. 2d).

### Factors controlling expression of the miR-29 family

Having shown the miR-29 family to be expressed in models of OA and cartilage development and regulated directly by Sox9, we explored the regulation of the miR-29 family in chondrocytes by cytokines and growth factors known to be important in cartilage homeostasis and OA.

**Fig. 3:**
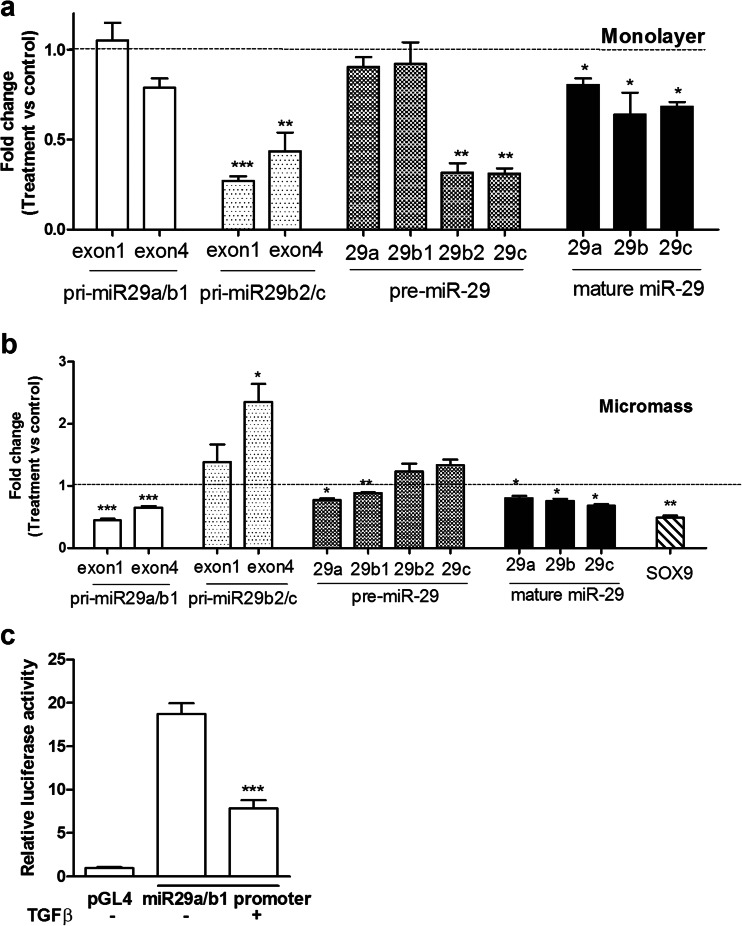
Regulation of the miR-29 family by TGFβ1. Human primary articular chondrocytes were grown either in monolayer (**a**) or micromass (**b**) culture (as Materials and Methods). In monolayer, cells were transferred to 0.5 % (*v*/*v*) FCS-containing medium overnight before stimulation with TGFβ1 (4 ng/ml) or vehicle for 24 h. In micromass, cells were stimulated for 48 h with TGFβ1 (10 ng/ml) or vehicle in 10 % (*v*/*v*) FCS-containing medium. Expression of the primary miR-29a/b1, -29b2/c, precursor miR-29a, -29b1, -29b2, -29c and the mature miR-29a, b, c or SOX9 were measured by qRT-PCR. Primers from two regions of the primary miRNA were used as shown (exon 1 and exon 4). Data were normalized to 18S rRNA expression for primary and precursor miRNA and to U6 expression for mature miR-29 and then vehicle control (mean ± SEM, ANOVA, *n* = 3). *Empty bars*, pri-miR29a/b1; *light grey bars*, pri-miR29-b2/c; *dark grey bars*, pre-miR-29; *black bars*, mature miR-29; *horizontal line at 1*, vehicle control. A primary miR-29a/b1 promoter-reporter construct or empty pGL4 vector (**c**) were transfected into SW1353 cells; after serum starvation for 24 h, cells were stimulated with TGFβ1 (4 ng/ml) or vehicle for a further 6 h before measuring luciferase activity. Data were normalised to Renilla luciferase from a co-transfected constitutive expression vector (mean ± SEM, ANOVA, *n* = 6). *p < 0.05; **p < 0.01; ***p < 0.001

Transforming growth factor beta (TGFβ) is a key factor in OA and has been shown to regulate miR-29 expression in fibrosis [18]. The impact of TGFβ1 on miR-29 expression in chondrocytes was investigated in monolayer culture and in three-dimensional micromass culture. Figure 3a shows that in monolayer culture, the primary and precursor transcripts of miR-29a and miR-29b1 were not regulated by TGFβ1, though the mature miR-29a-3p was still reduced. Expression of primary miR-29b2/c, the precursor and mature miR-29b and miR-29c were significantly repressed by TGFβ1 in monolayer culture. In micromass culture, the converse was true (Fig. 3b), with no effect (or an increase) on primary miR-29b2/c or precursors for miR-29b2 and miR-29c, though the mature miR-29c-3p was still reduced. Expression of primary miR-29a/b1 and the precursor and mature miR-29a-3p and miR-29b-3p were significantly repressed by TGFβ1 in micromass culture. This suggests not just regulation of transcription, but also at the level of maturation of the precursor to mature form of the microRNA by TGFβ1. In a transient transfection experiment, the miR-29a/b1 promoter is repressed by TGFβ1 in SW1353 cells (Fig. 3c).

**Fig. 4:**
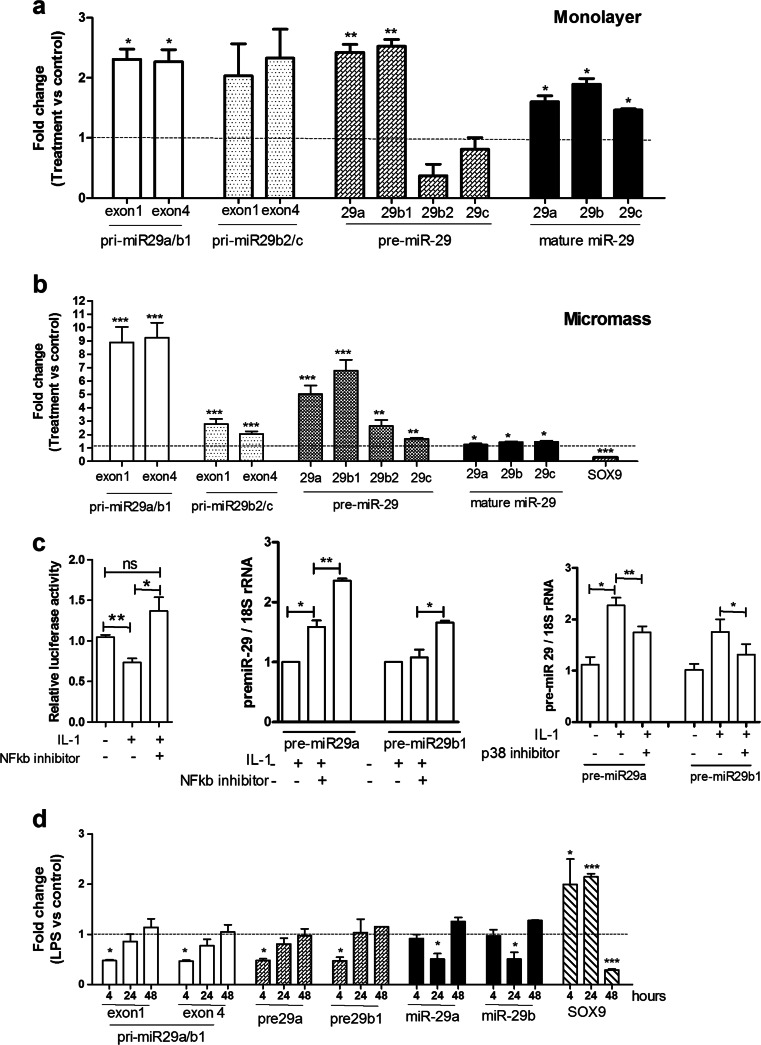
Regulation of the miR-29 family by interleukin-1 and LPS. Human primary articular chondrocytes were grown either in monolayer (**a**) or micromass (**b**) culture (as Methods). In monolayer, cells were transferred to 0.5 % (*v*/*v*) FCS-containing medium overnight before stimulation with IL-1β (5 ng/ml) or vehicle for 24 h. In micromass, cells were stimulated for 48 h with IL-1β (10 ng/ml) or vehicle in 10 % (*v*/*v*) FCS-containing medium. Expression of the primary miR-29a/b1, -29b2/c, precursor miR-29a, -29b1, -29b2, -29c and the mature miR-29a, b, c or SOX9 were measured by qRT-PCR. Primers from two regions of the primary miRNA were used as shown (exon 1 and exon 4). Data were normalized to 18S rRNA expression for primary and precursor miRNA and to U6 expression for mature miR-29 and then vehicle control (mean ± SEM, ANOVA, *n* = 3). *Empty bars*, pri-miR-29a/b1; *light grey bars*, pri-miR-29b2/c; *dark grey bars*, pre-miR-29; *black bars*, mature miR-29, *horizontal line at 1*, vehicle control. **c** A primary miR-29a/b1 promoter-reporter construct was transfected into SW1353 cells; after serum starvation for 24 h, cells were stimulated with IL-1β (5 ng/ml), +/− an NFκB inhibitor JSH-23 (10 μM) or vehicle for a further 6 h before measuring luciferase activity. Data were normalised to Renilla luciferase from a co-transfected constitutive expression vector (mean ± SEM, ANOVA, *n* = 6). Human primary articular chondrocytes in monolayer were serum starved for 24 h and stimulated with IL-1β (5 ng/ml) +/− an NFκB inhibitor JSH-23 (10 μM) or a p38 MAPK inhibitor SB203580 (10 μM) or vehicle control for 6 h. Expression of pre-miR-29a and b1 were measured by qRT-PCR and normalized to 18S rRNA (mean ± SEM, ANOVA, *n* = 3). **d** Human primary articular chondrocytes were cultured in micromass as (**b**) and stimulated across 48 h with LPS (1 μg/ml) or vehicle in 10 % (*v*/*v*) FCS-containing medium (F); primary miR-29a/b1, precursor miR-29a, -29b1 and the mature miR-29a, b were measured as (**b**) (mean ± SEM, ANOVA, *n* = 3). *p < 0.05; **p < 0.01; ***p < 0.001. *Empty bars*, pri-miR-29a/b1; *dark grey bars*, pre-miR-29; *black bars*, mature miR-29; *horizontal line at 1, *vehicle control

Interleukin-1 (IL-1) is a catabolic cytokine, implicated in destruction of cartilage in OA [34]. In monolayer culture, IL-1 increased expression of the primary miR-29a/b1 transcript, precursors of miR-29a and miR-29b1, and the mature miR-29a-3p and miR-29b-3p (Fig. 4a). No significant induction of pri-miR-29b2/c or pre-miR-29b2 or pre-miR-29c was seen, though IL-1 increased the mature miR-29c-3p (Fig. 4a). In micromass culture, IL-1 increased expression of all primary and precursor transcripts and the mature miR-29 family (Fig. 4b). Interestingly, the miR-29a/b1 promoter was repressed by IL-1, and this was rescued by inhibiting NFκB with JSH-23 (Fig. 4c). Similarly, the induction of miR-29 precursors was also augmented by inhibiting NFκB (Fig. 4c), suggesting that NFκB acts as a negative regulator of miR-29 expression. LPS, another factor which induces NFκB, decreased the expression of pri- and pre-miR-29a and miR-29b1 at an early time point (4 h), with the mature miR-29a-3p and miR-29b-3p reduced at 24 h (Fig. 4d). A similar pattern was seen for miR-29b2 and miR-29c, though this did not reach significance (data not shown). The miR-29a/b1 promoter was regulated by LPS in an identical manner to IL-1 (data not shown), with suppression rescued by an NFκB inhibitor. A p38 inhibitor (SB203580) blocked induction of the miR-29 family by IL-1, showing that this was, at least in part, dependent on the p38 pathway (Fig. 4c).

The Wnt pathway has also been implicated in OA [35]. However, microRNA-29 expression was not modulated by Wnt3a with no effect on the miR-29a/b1 promoter (data not shown).

There is no simple relationship between regulation of SOX9 by these factors and that of miR-29. For example, in micromass, TGFβ1 decreases SOX9 by 2.0-fold (Fig. 3b), IL-1 decreases SOX9 by 3.2-fold (Fig. 4b) and LPS increases SOX9 by 2.0-fold at 4 h, 2.15-fold at 24 h and decreases it by 3.5-fold at 48 h (Fig. 4d). Whilst this shows a correlation with miR-29 expression for IL-1, this is not true for TGFβ1 and LPS. Wnt3a also decreases SOX9 expression by approximately threefold, yet has no effect on miR-29 expression (data not shown).

### The miR-29 family regulates key signalling pathways

In order to probe intracellular signalling pathways which are involved in OA, constructs were used in which luciferase expression was driven by specific factors: p(CAGA)_12_-luc which responds to Smad2/3/4 as a readout of TGFβ1 activation; κB-luc which responds to NFκB as a readout of IL-1 or LPS activation; TOPFlash which responds to canonical Wnt signalling. These were reinforced by measuring the expression of genes which respond to these agonists: *ADAMTS4* as a TGFβ1-inducible gene; *MMP3* as an IL-1-inducible gene and *Axin2* as a Wnt-inducible gene.

**Fig. 5:**
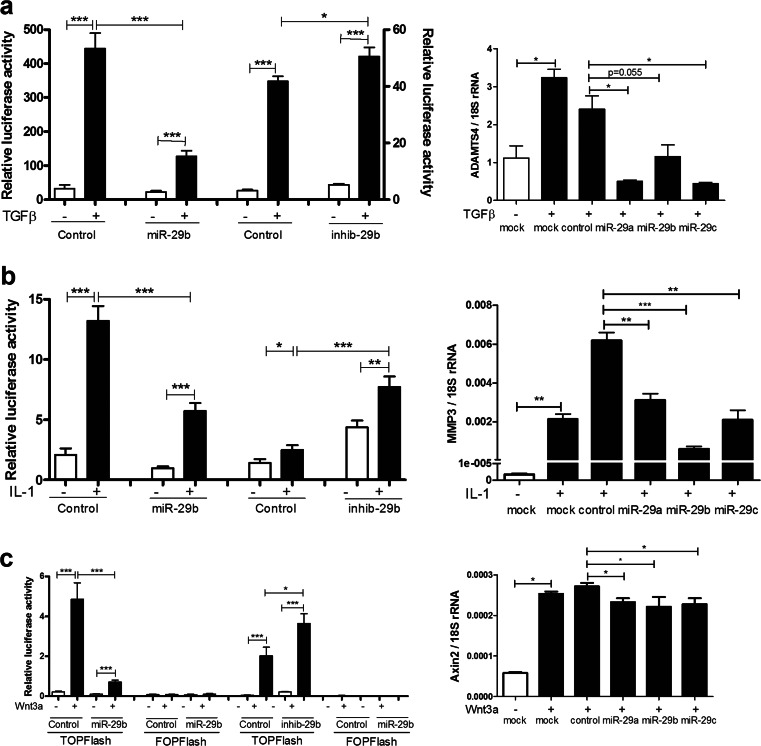
Regulation of intracellular signaling pathways by miR-29b-3p. SW1353 cells were transfected with (CAGA)12-luc (**a**), κB-luc (**b**) or TOPFlash and FOPFlash vectors (**c**) and co-transfected with either 50 nM miR-29b-3p mimics, inhibitors or non-targetting controls. After 24 h, cells were serum starved and treated with TGFβ1 (4 ng/ml) (**a**), IL-1β (5 ng/ml) (**b**) or Wnt3a (100 ng/ml) (**c**) for 6 h prior to assay for luciferase activity. Data were normalized to Renilla luciferase (mean ± SEM, Student’s *t* test, *n* = 6). Human primary chondrocytes were transfected with miR-29-3p family mimics or non-targeting control (50 nM) for 24 h, after culture in low serum (0.5 % *v*/*v* FCS) for 24 h, cells were stimulated with TGFβ1 (4 ng/ml) (**a**), IL-1β (5 ng/ml) (**b**) or Wnt3a (100 ng/ml) (**c**) for 24 h. Gene expression (ADAMTS4 **a**; MMP3 **b**; Axin2 **c**) was measured by qRT-PCR and normalized to 18S rRNA (mean ± SEM, ANOVA, *n* = 3). *p < 0.05; **p < 0.01; ***p < 0.001. *Empty bars*, control; *black bars*, treatment

Since TGFβ1 regulated expression of the miR-29 family, we measured the Smad 2/3/4 pathway using transient transfection of the p(CAGA)_12_-luc Smad-responsive plasmid. Figure 5a shows that a miR-29b-3p mimic repressed Smad signalling, inhibiting the TGFβ1 induction of the construct; conversely, an inhibitor of miR-29b-3p augmented TGFβ1 induction. Figure 5a shows that TGFβ1-induced ADAMTS4 expression was repressed by transfection of miR-29 family mimics, verifying the effect on an endogenous gene.

Similarly, miR-29-3p repressed NFκB signalling, inhibiting IL-1 induction of a κB-luc construct, whilst the inhibitor of miR-29b-3p augmented the response (Fig. 5b). The MMP3 gene, which is responsive to NFκB, as well as other transcription factors, was induced by IL-1 and significantly repressed by miR-29 family mimics (Fig. 5b).

Whilst miR-29 expression is not regulated by Wnt3a, we measured the impact of miR-29 on canonical Wnt signalling, using the TOPFlash construct. The miR-29-3p mimic repressed Wnt3a induction of this construct (with no effect on the control FOPFlash), whilst it was augmented by the miR-29b-3p inhibitor (Fig. 5c). Wnt3-induced Axin2 expression was significantly, though minimally, repressed by miR-29 family mimics (Fig. 5c).

### The action of the miR-29 family in articular chondrocytes

**Fig. 6:**
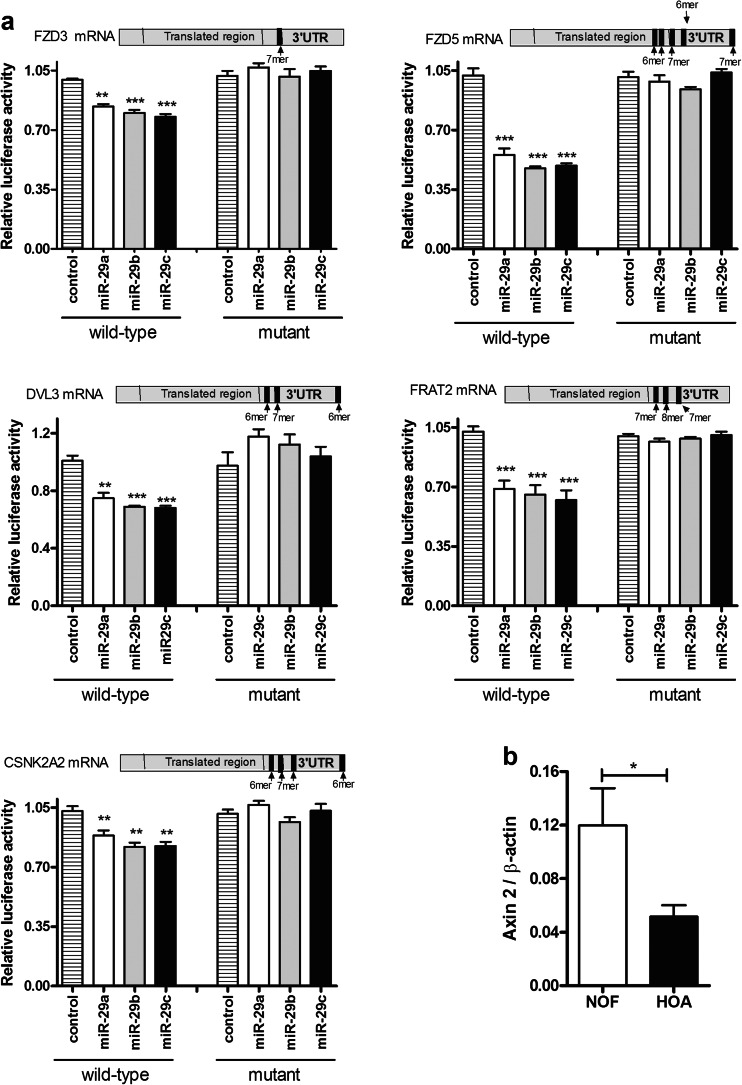
The miR-29 family directly targets members of the Wnt pathway. **a** The 3’ UTR of *FZD3*, *FZD5*, *DVL3*, *FRAT2* and *CSNK2A2* was subcloned downstream of luciferase in the pmiRGLO vector to create the wild-type construct. All seed sites of the miR-29 family were altered to non-binding sequences (number of sites shown) to create mutant constructs. Constructs were transfected into DF1 fibroblasts with miR-29 mimics or non-targeting controls (50 nM) and assayed for luciferase activity after 24 h. Data were normalised to Renilla luciferase and then the non-targeting control (mean ± SEM, ANOVA, *n* = 6). *Striped bars*, control; *empty bars*, miR-29a; *grey bars*, miR-29b; *black bars*, miR-29c. **b** Expression of axin2 was measured in cartilage from hip osteoarthritis (OA) patients compared to fracture (NOF) controls (qRT-PCR, mean ± SEM, Student’s *t* test, *n* = 12)

We performed gain-of-function and loss-of-function experiments to identify miR-29 targets in primary HACs. Cells were transfected with miR-29b-3p mimics, inhibitors or non-targeting controls, and the transcriptome analysed on Illumina microarray. Our previous research [36] showed that miRNA targets are enriched amongst genes which are decreased upon overexpression of the miRNA, increased on inhibition of the miRNA and contain seed sites for the miRNA. This intersection contained a number of known direct targets for miR-29 (e.g. COL1A1 and COL3A1), see Supplementary Table 3. Pathway analysis (DAVID) highlighted the Wnt signalling pathway, including DVL3, (Dishevelled 3); CSNK2A2, (casein kinase 2 alpha 2 polypeptide); FRAT2, (Frequently Rearranged In Advanced T-Cell Lymphomas 2, a GSK-3 binding protein); FZD3, (Frizzled family receptor 3) and FZD5, (Frizzled family receptor 5). The 3’ UTR of all these genes was cloned into a luciferase reporter and co-transfection of the miR-29-3p mimics resulted in significant repression of luciferase expression (Fig. 6a). Mutation of miR-29 seed sites (1–5 sites, depending on gene), abolished this repression, demonstrating that all these genes are direct targets of the miR-29 family (Fig. 6a). Interestingly, Axin2 expression, readout of canonical Wnt signalling, was decreased in hip OA cartilage compared to NOF (Fig. 6b) where miR-29 expression was increased (Fig. 1d).

## Discussion

This study was driven by expression profiling miRNAs at early time points post-surgery in the murine DMM model of OA. Using whole knee joint RNA, only two miRNA altered significantly in expression 24 h after surgery and of these, miR-29b-3p was inversely correlated with expression of its putative targets. Further, expression of the miR-29 family was significantly increased in human cartilage from end-stage OA. Expression of miR-140-5p is also increased in OA in these samples, despite published data showing the converse and the increased susceptibility to OA in the miR-140 null mouse [8, 14]. This may reflect stage of the disease and/or the control tissue (see, e.g. [14]). Guerit et al. [37] show no difference in expression of miR-29a in OA compared to healthy cartilage, but the source of the tissue is uncertain. Since expression of the miR-29 family is increased immediately post-surgery in the DMM model and is increased upon hip cartilage avulsion, this may be a response to injury, a known phenomenon for miRNAs in several areas of physiology and pathology, including cartilage, e.g. [38, 39]. It is moot if the same mechanism(s) increase expression of the miR-29 family in cartilage from end-stage human OA.

The miR-29 family was regulated in both human and mouse models of cartilage development though decreased expression of the miR-29 family in models of chondrogenesis has been described before [37, 40, 41]. Expression of the miR-29 family was highest in human articular cartilage tissue, decreasing with cell passage in monolayer culture. This is opposite to the expression of COL1A1, a gene which it is known to regulate.

Since expression of the miR-29 family in the MSC chondrogenesis model was inversely correlated with expression of SOX9, we examined the role of SOX9 in the regulation of miR-29 expression. This showed SOX9 as a negative regulator of miR-29 expression, and we further demonstrated that this was via the promoter for the miR-29a/b1 cluster. This agrees with recent data from Guerit et al., with miR-29a decreasing across 21 days in their model of chondrogenesis and SOX9 increasing across 3 days and decreasing thereafter. They showed SOX9 negatively regulating miR-29 which allowed an increase in FOXO3A leading to the differentiation of MSC into chondrocytes [37]. Our data also show that there is no direct relationship between SOX9 and growth factor regulating the miR-29 family, in agreement with studies showing SOX9-dependent and independent regulation of gene expression by growth factors [42].

Recent research is unravelling the intracellular signalling pathways which underlie osteoarthritis and their interaction (e.g. [43]). It is clear that several pathways are involved in cartilage homeostasis and osteoarthritis and that their interaction is key to their phenotypic outcome [44, 45]. The NFκB pathway has a catabolic role in cartilage destruction, but pathways such at TGFβ and Wnt can have positive or negative effects on the tissue [46]. We pursued the relationship between these three factors and the miR-29 family in primary HACs since the literature suggests that this is cell-type specific.

TGFβ1 repressed expression of pri-miR-29b2/c and subsequent pre-miR-29b2 and c in monolayer culture, with no significant effect on the pri-miR-29a/b1 locus. Conversely, in micromass culture TGFβ1 repressed the pri-miR-29a/b1 locus, but not the pri-miR-29b2/c locus. There is a difference in culture conditions (e.g. level of FCS, time) between monolayer and micromass which may impact on this, but it is important to note that TGFβ1 effects in the three dimensional system are different. However, in both cases, all three mature members of the miR-29 family were repressed. TGFβ1 is known to repress the miR-29 family in a number of cell types (e.g. [18, 47], and this is the same in primary chondrocytes. Whilst this has been shown to be Smad3-dependent in, e.g. renal fibrosis [47], and our data agree with an effect on the promoter, it may be that processing from pre-miRNA to mature miRNA is the major determinant in their final expression controlled by TGFβ, rather than transcription. Previous reports have shown that a number of mature miRNA are induced by TGFβ1 at the level of DROSHA-mediated miRNA maturation [48], but no mechanism for TGFβ1 repression of miRNA maturation has been described. However, regulation of miRNA biogenesis by a number of factors has been described at every level [49]. MicroRNA-29 also represses TGFβ1-induced Smad signalling shown through the (CAGA)_12_-luc construct or endogenous TGFβ1 responsive genes in primary chondrocytes. This gives a feed forward loop where TGFβ1 reduces levels of miR-29 which are repressing the Smad pathway and therefore allows a greater increase in Smad-dependent gene regulation.

The NFκB pathway is repressed by miR-29 though there are opposing effects on miR-29 expression regulated by either IL-1 or LPS. Whilst LPS is not directly involved in OA, it is a TLR4 agonist which induces NFκB, and this pathway is strongly implicated in disease (e.g. [50, 51]). IL-1 induces the miR-29 family in a p38 MAPK-dependent manner, though the NFκB pathway represses miR-29 expression. This latter can be seen through LPS repression of miR-29 expression, but also an increased expression of miR-29 upon treatment with an NFκB inhibitor, both pre-miR-29 and at the level of the pri-miR-29a/b1 promoter. Expression of either p50, p65 or both together repressed the pri-miR-29a/b1 promoter (data not shown). In other cell lines, NFκB has been shown to act as an inhibitor of miR-29 [52–54].

Whilst Wnt3a does not regulate miR-29 expression in primary HACs, miR-29 can repress canonical Wnt signalling, on the TOPFlash construct and the Axin2 gene. Axin2 gene expression in osteoarthritic hip cartilage compared to a fracture control is inversely correlated with that of miR-29 in the same tissue. Negative regulation of canonical Wnt signalling by miR-29 has been shown to be via targeting of DNMT3A and 3B to demethylate WIF-1 in non-small-cell lung cancer [55]. In human osteoblasts, miR-29 is induced by canonical Wnt signalling and miR-29 potentiates Wnt signalling, demonstrating the cell type-specific nature of miR-29 function [27, 56]. Interestingly, miR-29 gain- and loss-of-function microarray experiments in primary HACs highlighted a number of genes from the Wnt signalling pathway as potential targets of miR-29 in articular chondrocytes. Several of these have been validated, showing the functional capability of miR-29 on this pathway in cartilage. FZD3 and FZD5 are Wnt receptors with DVL3 a downstream intracellular signalling molecule in the Wnt pathway. FRAT2 is a GSK3 binding protein and CSNK2A2 encodes a subunit of casein kinase 2 alpha [57, 58]. These are positive regulators of the Wnt pathway, and targeting them is likely to suppress Wnt signalling, which is the outcome of miR-29 action (Fig. 5).

**Fig. 7:**
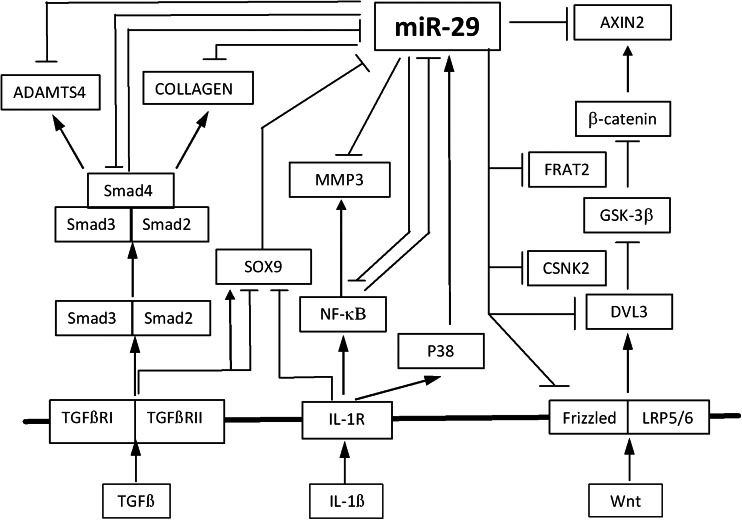
A schematic of the role of the miR-29 family in cartilage. Data from this study have identified a complex interplay between the miR-29 family and key signalling pathways which regulate cartilage homeostasis. The TGFβ, IL-1, and Wnt pathways regulate and/or are regulated by the miR-29 family highlighting a nonlinear system, with outcomes on the expression of matrix or matrix-degrading genes

These data show a complex role for the miR-29 family in cartilage homeostasis and OA. Figure 7 shows the relationships, we have identified amongst cytokines, growth factors and signalling pathways with the miR-29 family. These are not linear relationships, but rather result in complex feedback mechanisms which will regulate signalling in chondrocytes during development and disease. This figure demonstrates that the miR-29 family adds an additional level of regulation across key pathways of cartilage homeostasis, and its dysregulation could either lead or contribute to disease.

In summary, we have shown that the miR-29 family has a role in osteoarthritis, including early disease. The miR-29 family is regulated by a number of factors known to be important in OA and has a functional impact on several relevant signalling pathways. In these data, we have identified and validated a number of genes as new direct targets of the miR-29 family.

## Electronic supplementary material

Below is the link to the electronic supplementary material.

ESM 1 (PDF 637 kb)
