# Supplementary material for: The microRNA-29 family in cartilage homeostasis and osteoarthritis
Source: J Mol Med (Berl). 2015 Dec 19;94:583–96. doi: 10.1007/s00109-015-1374-z (PMC4856728; doi:10.1007/s00109-015-1374-z)
Supplement: Supplementary file 1 — (PDF 637 kb) [file 109_2015_1374_MOESM1_ESM.pdf]

Electronic Supplementary Materials

J Mol Med 2015

The microRNA-29 family in cartilage homeostasis and osteoarthritis

Linh T T Le, Tracey E Swingler, Natalie Crowe, Tonia L Vincent, Matthew J Barter, Simon T Donell, Anne M Delany, Tamas Dalmay, David A Young, Ian M Clark.

Correspondence to: Ian M Clark, School of Biological Sciences, University of East Anglia, Norwich, UK. i.clark@uea.ac.uk,

Supplementary Table 1: Primer sequences. For subcloning of 3'UTR, restriction sites (bases underlined) were added to the primers: *Sac*I (GAGCTC), *Sal*I (GTCGAC), *Xba*I (TCTAGA).

Gene	Forward primer	Reverse primer	Probe (if applicable)
ADAMTS4	5'-CAAGGTCCCATGTGCAACGT-3'	5'-CATCTGCCACCACCAGTGTCT-3'	5'- FAM-CCGAAGAGCCAAGCGCTTTGCTTC-TAMRA-3'
MMP3	5'-TTCCGCCTGTCTCAAGATGATAT-3'	5'-AAAGGACAAAGCAGGATCACAGTT-3'	5'-FAM-TCAGTCCCTCTATGGACCTCCCCCTGAC-TAMRA-3'
SOX9	5'-TACCCGCACTTGCACAAC-3'	5'-TCTCGCTCTCGTTCAGAAGTC-3'	Probe #61 (Roche Universal probe library)
AXIN2	5'-GCTGACGGATGATTCCATGT-3'	5'-ACTGCCCACACGATAAGGAG-3'	Probe #56 (Roche Universal probe library)
Pri-miR-29a/b1exon 1	5'-TACTGAACTGTCACGGCAGA-3'	5'-TGTAGTTAGCGACCTCTGCT-3'	n/a SYBR green
Pri-miR-29a/b1Exon 4	5'-TTGCACCCTCACGACATGCT-3'	5'-TGACTCTCAGCAGGCCTCA-3'	n/a SYBR green
Pri-miR-29b2/c exon 1	5'-ACTTCTTTAGGGGTGTGCGTA-3'	5'-ACCCATCTCCCTAGCATTCT-3'	n/a SYBR green
Pri-miR-29b2/c Exon6	5'-TCAGACTTGCCACCTGGACT-3'	5'-AGTTGGCATGAGGCTTCGA-3'	n/a SYBR green
Pre-29a	5'-CTGATTTCTTTTGGTGTTCAG-3'	5'-AACCGATTTCAGATGGTGC-3'	n/a SYBR green
Pre-29b1	5'-CATATGGTGGTTTAGATTT-3'	5'-AACACTGATTTCAAATGGT-3'	n/a SYBR green
Pre-29b2	5'-GCTGGTTTCACATGGTGGC-3'	5'-AACACTGATTTCAAATGGTG-3'	n/a SYBR green
Pre-29c	5'-CGATTTCTCCTGGTGTTCA-3'	5'-ACCGATTTCAAATGGTGC-3'	n/a SYBR green
ADAMTS6	5'-ACGTGAGCTCTCTCATCGTCATGGTTCTGC-3'	5'-ACGTGAGCTCCAAGCAGGAGAATGAATGTAGG-3'	
ADAMTS6 (1st seed site)	5'-TATGTGATGCACTGACATGTAATTTAAGAAGCTTATGATGGAATCAAGTCAAACATGCTGTTTAACTGAAAG-3'	5'-CTTTCAGTTAAACAGCATGTTTGACTTGATTCCATCATAAGCTTCTTAAATTACATGTCAGTGCATCACATA-3'	
ADAMTS6 (2nd seed site)	5'-TATTTATTTCACCAGGGCACATTAAGCTTAAGTTAACTGTTCTTTGAAAAGGCGCAAGGGAATTCAGT-3'	5'-ACTGAATTCCCTTGCGCCTTTTCAAAGAACAGTTAACTTAAGCTTAATGTGCCCTGGTGAAATAAATA-3'	
ADAMTS10			
ADAMTS10 (1st seed site)	5'-GGGGACACAGACCCGTTTGTAAGCTTACCCCTTGTCGATGGTGTGCG-3'	5'-CGCACACCATCGACAAGGGGTAAGCTTACAAACGGGTCTGTGTCCCC-3'	
ADAMTS10 (2nd seed site)	5'-GCTCGGTCCGGGCCAAGCTTATGACGATGAGAGATGCATTAATCGGTCC-3'	5'-GGACCGATTAATGCATCTCTCATCGTCATAAGCTTGGCCCGGACCGAGC-3'	

ADAMTS14	5’-ACTGGAGCTCGCTGTGCCCTGCCATC-3’	5’-ACTGGAGCTCGGGTCCAATGGCGATGTTA-3’
ADAMTS14 (1st seed site)	5’-GTTTGTCTTTGCTGGCCAGAAGAGTCGACTCATGGCCATACTCTGGCCTTG-3’	5’-CAAGGCCAGAGTATGGCCATGAGTCGACTCTTCTGGCCAGCAAAGAC-3’
ADAMTS14 (2nd seed site)	5’-GGGTGCCAGCCCCTGGCCGTCGACTGGAGTGGGGAAGACAC-3’	5’-GTGTCTTCCCCACTCCAGTCGACGGCCAGGGGCTGGCACCC-3’
ADAMTS14 (3rd seed site)	5’-CTAAACTCCTGCCAGGTGATAGAGAGCTCTCTCACTTCTTCCTTCCCCAAGGC-3’	5’-GCCTTGGGGAAGGAAGAAGTGAGAGAGCTCTCTATCACCTGGCAGGAGTTTAG-3’
ADAMTS14 (4th seed site)	5’-CTAAACTCCTGCCAGGTGATAGAGAGCTCTCTCACTTCTTCCTTCCCCAAGGC-3’	5’-GCCTTGGGGAAGGAAGAAGTGAGAGAGCTCTCTATCACCTGGCAGGAGTTTAG-3’
ADAMTS17	5’-ACGTTCTAGAAACATGAGCGTGGACTTGG-3’	5’-ACGTTCTAGATGTAATGCAAGTTAACGAATGG-3’
ADAMTS17 (1st seed site)	5’-GCAATTACCGTTTCTTATGTCACAGTCGACTGAAGAGAGGCCCTTCTGTTTCCC-3’	5’-GGGAAACAGAAGGGCCTCTCTTCAGTCGACTGTGACATAAGAAACGGTAATTGC-3’
ADAMTS17 (2nd seed site)	5’-CACCAACTTGGTGGGCATTTCATGTCGACTTATGTTCTAGGACTTTACCGTA-3’	5’-TACGGTAAAGTCCTAGAACATAAGTCGACATGAAATGCCCACCAAGTTGGTG-3’
ADAMTS17 (3rd seed site)	5’-TAACAAAACAAAACACAGAAACACAGTCGACATAAATCAAGAAGCACAGGGAGATGATCCCATGG-3’	5’-CCATGGGATCATCTCCCTGTGCTTCTTGATTTATGTCGACTGTGTTTCTGTGTTTTGTTTTGTTA-3’
ADAMTS17 (4th seed site)	5’-GAAGTGTTGAGAAACTTCCGTGTCGACTCTGTGGAAAGAACCGAGGGT-3’	5’-ACCCTCGGTTCTTTCCACAGAGTCGACACGGAAGTTTCTCAACACTTC-3’
ADAMTS17 (5th seed site)	5’-CCAGAGTCTCACGACCCTACGGTCGCCTTTTTATTGGTGCAAAATTAAACC-3’	5’-GGTTTAATTTTGCACCAATAAAAAGGCGACCGTAGGGTCGTGAGACTCTGG-3’
ADAMTS19	5’-ACGTGAGCTCAATCACAGCTCCAGGTAATC-3’	5’-ACGTGAGCTCCCAAGAGACATACTATCTTCCAAGG-3’
ADAMTS19 (1st seed site)	5’-ATCAAATTAATTTATTTTTTTGCCTGCCAAACATCCAATGGTCGACTTGTTTTGGTTACACAAACATTTTGATTTATACTATATG-3’	5’-CATATAGTATAAATCAAAATGTTTGTGTAACCAAAACAAGTCGACCATTGGATGTTTGGCAGGCAAAAAAATAAATTAATTTGAT-3’
ADAMTS19 (2nd seed site)	5’-GTTGTTTGTTAGGGCTATCTCTAAGTCGACCCTCTCTCCCCACCAATAACATTGAATTATC-3’	5’-ATAATTCAATGTTATTGGTGGGGAGAGAGGGTCGACTTAGAGATAGCCCTAACAAACAACG-3’
FZD3	5’-ATGCGTCGACTATTAGATGCCCAGCCTTTCTC-3’	5’-ATGCGTCGACATGCCTACCAAGAGGATAACATTC-3’
FZD3 (1st seed site)	5’-GGATTTAGTCTAACTCACAGCTAAGGTAGAAAAGTACTCTGATGGCAAGAGAATGTCCAGACTAATATTTTC-3’	5’-GAAAATATTAGTCTGGACATTCTCTTGCCATCAGAGTACTTTTCTACCTTAGCTGTGAGTTAGACTAAATCC-3’

FZD5	5’-ATGCGTCGACGGCATCGGCTACAACCTGAC-3’	5’-ATGCGTCGACAGACCACACAGTTCAAAGA AACCTG-3’
FZD5 (1st seed site)	5’-CGGCGTCGCGGCCCAAGCTTGGGAGGCGGTCGCAG-3’	5’-CTGCGACCGCCTCCCAAGCTTGGGCCGCGACGCCG-3’
FZD5 (2nd seed site)	5’-GTGGACGTGGAGATGAAGCACAAGCTTGACCACAGGCCTATCCAGAAGG-3’	5’-CCTTCTGGATAGGCCTGTGGTCAAGCTTGTGCTTCATCTCCACGTCCAC-3’
FZD5 (3rd seed site)	5’-GCCCACCAGCAGGTAGAAGCTTAGCGGGCCCAGCACGAAGCC-3’	5’-GGCTTCGTGCTGGGCCCGCTAAGCTTCTACCTGCTGGTGGGC-3’
FZD5 (4th seed site)	5’-CACATGAAGTACTTGAGCATGAAGCTTCAGTACTCGGGCTTGGCGCGCG-3’	5’-CGCGCGCCAAGCCCGAGTACTGAAGCTTCATGCTCAAGTACTTCATGTG-3’
FZD5 (5th seed site)	5’-CGGGAGGGGGCAACAAGCTTATGAAGGTAAACGGAAGTGACCTTGGCA-3’	5’-TGCCAAGGTCACTTCCGTTTACCTTCATAAGCTTGTTGCCCCCTCCCG-3’
FRAT2	5’-ATGCGTCGACCAACAGCGTCCAGTTCCTAC-3’	5’-ATGCGTCGACGCCGTCAAGTTTCATACAGC-3’
FRAT2 (1st seed site)	5’-GCGTGGAGAAATGTATGCGCCAGAAGCTTTCCGTGGGGCATGAGAATTTCC-3’	5’-GGAAATTCTCATGCCCCACGGAAAGCTTCTGGCGCATACATTTCTCCACGC-3’
FRAT2 (2nd seed site)	5’-CTTATTTTCTGGTGGAGGAGCTTAGTAAGTAAGCTTACAATTGCTGTGCAAAGAAATTCCAGAGG-3'	5’-CCTCTGGAATTTCTTTGCACAGCAATTGTAAGCTTACTTACTAAGCTCCTCCACCAGAAAATAAG-3’
FRAT2 (3rd seed site)	5’-GGGAGACTCCAAGCGGTGGTAAAAGCTTAACAGGGCTCTTCTTGGAGCAAG-3’	5’-CTTGCTCCAAGAAGAGCCCTGTTAAGCTTTTACCACCGCTTGGAGTCTCCC-3’
CK2A2	5’-ATGCGTCGACATGCAGGTACTAGAGTTGTGTGG-3’	5’-ATGCGTCGACAATAAGTTTGCTTGTTTCTGTGG-3’
CK2A2 (1st seed site)	5’-AGAGGAATATACAAGGGGCTTGGGGAAGAAAATAAGCTTCCCGGAGCAAGTGTTG-3’	5’-CAACACTTGCTCCGGGAAGCTTATTTTCTTCCCCAAGCCCCTTGTATATTCCTCT-3’
CK2A2 (2nd seed site)	5’-TCTCCTCTAATCTATCAGTCTGAGAAGCTTTTCCTCTCTGCAAGGGAACACATTTGC	5’-GCAAATGTGTTCCCTTGCAGAGAGGAAAAGCTTCTCAGACTGATAGATTAGAGGAGA
CK2A2 (3rd seed site)	5’-GCGCCTGACTCGAGAAGCTTACCTTTCAGTCCACTGGGACCAATCCA-3’	5’-TGGATTGGTCCCAGTGGACTGAAAGGTAAGCTTCTCGAGTCAGGCGC-3’
CK2A2 (4th seed site)	5’-CTGCTTCCATCCTTATCAACAGAAGCTTTGGGAGAACCTAAGTCATTTCCCTGAG-3’	5’-TCAGGGAAATGACTTAGGTTCTCCCAAAGCTTCTGTTGATAAGGATGGAAGCAG-3’
DVL3	5’-ATGCGTCGACGCTGCGTTCCTCTCTCCATC-3’	5’-ATGCGTCGACTACCATTTATTGAGCACCTACTCTACTGTG-3’
DVL3 (1st seed site)	5’-GTGCGCTAACTGCTCGCAGAAGCTTGCGAGGGTGGGGTGCACC-3’	5’-GGTGCACCCCACCCTCGCAAGCTTCTGCGAGCAGTTAGCGCAC-3’

DVL3 (2nd seed site)	5'-CCCTTTTGTCTCTGGGACCAGACTTGTTAAGCTTACCCCTTACTCCCCTCTGC-3'	5'-GCAGAGGGGAGTAAGGGGTAAGCTTAACAAGTCTGGTCCCAGAGACAAAAGGG-3'
DVL3 (3rd seed site)	5'-GCACAGTGCCTGGCACACAGTAGAGTAAAGCTTCAATAAATGGTAGTCGACC-3'	5'-GGTCGACTACCATTTATTGAAGCTTTACTCTACTGTGTGCCAGGCACTGTGC-3'

Supplementary Table 2: MicroRNAs regulated in the DMM model (operated right knee compared to un-operated left knee) at 1, 3 and 7 days post-surgery with a fold change greater than 1.5 (higher or lower). Down-regulated microRNAs are represented as negative fold change.

	miRNA	Fold change
Day 1	miR-144-3p	1.7
	miR-29b-3p	1.5
Day 3	miR-370-5p	1.7
	miR-21-5p	1.6
Day 7	miR-379-5p	2.6
	miR-127-3p	2.4
	miR-335-5p	2.4
	miR-370-5p	2.2
	miR-214-3p	2.2
	miR-21-5p	2.1
	miR-3073-3p	2
	miR-199a-3p	1.9
	miR-214-5p	1.8
	miR-210-3p	1.8
	miR-455-3p	1.8
	miR-199a-5p	1.7
	miR-2137	1.7
	miR-199b-5p	1.7
	miR-136-5p	1.7
	mir-34a-5p	1.6
	miR-99b-5p	1.6
	miR-152-3p	1.5
	miR-34c-5p	1.5
	miR-144-3p	-1.5
	miR-3100-3p	-1.5
	miR-669c-3p	-1.6
	miR-378-3p	-1.6
	miR-3473b	-1.6
	miR-133a-5p	-1.6
	miR-3474	-1.7
	miR-378b	-1.7
	miR-133a-3p	-1.8
	miR-133b-3p	-1.8
	miR-1952	-1.9
	miR-491-3p	-1.9
	miR-1a-3p	-2.2
	miR-706	-2.3
	miR-3572	-2.3

Supplementary Table 3: Candidate target genes of miR-29b which increase in expression when treated with microRNA inhibitor and decrease in expression with microRNA mimic

Gene	Mimic (fold change down)	Inhibitor (fold change up)	miR-29 target sites		
			6mer	7mer	8mer
COL1A1	2.53	1.69	3	3	1
C7orf41	2.33	1.21	2	1	0
PXDN	2.23	1.37	2	2	1
COL5A2	2.20	1.27	2	2	1
SH3PXD2A	2.16	1.15	4	3	1
GGCT	1.90	1.20	1	1	0
PSAT1	1.83	1.28	1	0	0
COL11A1	1.80	1.25	2	2	0
COL15A1	1.73	1.22	2	1	1
NDUFA10	1.71	1.33	2	1	0
H19	1.68	1.16	2	1	1
TP53INP1	1.68	1.38	1	1	1
ADAM19	1.64	1.28	6	2	0
MFAP2	1.59	1.24	2	2	0
P2RY6	1.58	1.26	1	0	0
WISP2	1.58	1.21	1	0	0
SPNS1	1.56	1.20	1	1	0
CCND2	1.52	1.20	2	2	0
VAMP7	1.47	1.16	2	1	0
PRMT6	1.45	1.18	1	1	0
KLHL24	1.45	1.21	2	1	0
SLC2A3	1.43	1.27	1	1	0
S100A16	1.43	1.18	2	2	0
EIF4E2	1.43	1.15	1	1	1
MEF2D	1.43	1.19	3	0	0
CDKN1A	1.43	1.15	1	0	0
FSTL1	1.41	1.24	2	1	0
C10orf54	1.41	1.17	6	2	0
PPIC	1.40	1.29	2	1	1
TOMM20	1.40	1.19	2	2	0
SPARC	1.39	1.15	3	2	0
TSPAN9	1.39	1.18	4	1	0
CADM1	1.38	1.29	1	1	0
GPX7	1.38	1.29	1	0	0
DYM	1.38	1.19	1	1	1
COL3A1	1.36	1.26	3	2	2
C5orf33	1.34	1.15	2	1	0
RNF144A	1.34	1.16	3	1	0
KLHDC3	1.33	1.31	1	1	0
SMS	1.33	1.19	1	1	0

COMMD2	1.32	1.20	2	1	1
ARHGAP28	1.32	1.24	1	0	0
LEPREL4	1.31	1.17	2	0	0
MGAT4B	1.30	1.26	1	1	0
EML4	1.30	1.21	2	1	0
SLC25A13	1.28	1.35	1	1	0
DIXDC1	1.28	1.16	2	1	0
JMY	1.27	1.19	3	1	0
PLEKHA1	1.27	1.33	1	1	0
PPP1R14C	1.27	1.22	2	0	0
PTGFR	1.26	1.21	1	0	0
PTGFR	1.26	1.18	1	0	0
TRIB3	1.26	1.40	3	1	0
GEMIN2	1.26	1.18	1	1	0
LAMC1	1.26	1.31	1	1	1
NPC1	1.25	1.20	1	0	0
SIRT1	1.25	1.18	1	1	1
AHNAK	1.25	1.26	1	1	0
WIPI1	1.24	1.16	1	0	0
VPS25	1.23	1.16	1	1	0
CREG1	1.23	1.19	2	2	0
SESTD1	1.22	1.33	2	2	2
AP3M2	1.22	1.17	1	1	0
COL4A1	1.22	1.41	2	2	1
TMEM176A	1.22	1.19	1	1	0
SUV420H1	1.22	1.19	1	1	0
LOC653994	1.22	1.17	3	2	0
AZIN1	1.22	1.39	1	0	0
PTDSS2	1.22	1.15	1	0	0
NNAT	1.22	1.16	1	0	0
CBX6	1.22	1.16	3	3	0
KDM5B	1.22	1.38	2	1	0
MAPK6	1.21	1.15	1	0	0
SGK1	1.21	1.20	1	1	1
